# Beyond league tables: a multidimensional and rank-aggregation assessment of Saudi universities under vision 2030

**DOI:** 10.3389/frma.2026.1993617

**Published:** 2026-10-30

**Authors:** Fouad Ahmed Atallah

**Affiliations:** Department of Sharia, College of Sharia and Law, Jouf University, Sakakah, Saudi Arabia

**Keywords:** ARWU, cross-ranking profile assessment, Pareto analysis, QS, rank aggregation, SDG 16, Saudi universities, Saudi Vision 2030

## Abstract

Global university rankings increasingly influence higher education policy and institutional benchmarking, yet major ranking systems differ substantially in their indicators, weighting, normalization, and aggregation procedures. This raises questions about the robustness of conclusions drawn from a single league table. The issue is particularly relevant to Saudi Arabia, where the Human Capability Development Program under Saudi Vision 2030 establishes targets for increasing the number of Saudi universities among the global top 100 and top 200 and references QS, Times Higher Education (THE), and Shanghai Ranking. This study examines the consistency of QS, THE, and the Academic Ranking of World Universities (ARWU) in representing Saudi universities over the aligned 2022–2025 period. Cross-ranking coverage and positional agreement were assessed using Jaccard overlap, Spearman correlation, and Kendall correlation. Consensus rankings were constructed using Borda, median-rank, and Copeland aggregation, complemented by cross-ranking profile and Pareto assessment and sensitivity analyses under alternative weighting and rank-band specifications. QS and THE generally showed stronger positional agreement, reaching a Spearman correlation of 0.932 in 2025, whereas QS-ARWU agreement in the same year was weaker (ρ = 0.437). The three aggregation procedures produced broadly concordant consensus structures, although the leading position was method-sensitive in 2025: Borda ranked King Saud University first, whereas median-rank and Copeland ranked King Fahd University of Petroleum and Minerals first. Cross-ranking profile analysis further showed that King Abdulaziz University, King Saud University, and King Fahd University of Petroleum and Minerals were simultaneously non-dominated on the Pareto frontier in 2024 and 2025, indicating that a single consensus ordering can conceal differences across ranking-system outputs. Policy-threshold analysis likewise produced different Top-100 and Top-200 counts and institutional identities across the three systems. Overall, QS, THE, and ARWU provided related but non-equivalent representations of university standing. Combining cross-ranking comparison, rank aggregation, and cross-ranking profile assessment can therefore support more transparent monitoring and interpretation of ranking-based institutional and policy outcomes under Saudi Vision 2030.

## 1 Introduction

Global university rankings have become influential instruments in higher education governance. They shape institutional reputation, student choice, international partnerships, strategic planning, and increasingly national policy. Their apparent simplicity, however, can obscure the methodological decisions through which institutional performance is converted into a single ordinal position. Indicator selection, data sources, normalization, weighting, and aggregation all influence the resulting league table. Rankings should therefore be understood not as neutral measurements of an underlying and universally defined institutional quality, but as evaluative frameworks that operationalize particular conceptions of university excellence (Moed, 2017; Latifis et al., 2026). This distinction becomes especially important when rankings are used not merely for information or comparison but as indicators of institutional and national policy performance.

The QS World University Rankings (QS), Times Higher Education World University Rankings (THE), and Academic Ranking of World Universities (ARWU) illustrate this methodological plurality. Although all three evaluate universities globally, they differ substantially in what they measure and how they combine their indicators. ARWU is predominantly research-oriented, emphasizing internationally recognized scientific achievement and research output; QS combines bibliometric measures with substantial reputational, employability, internationalization, and related indicators; and THE evaluates universities across teaching, research environment, research quality, international outlook, and industry. Consequently, the same university can occupy markedly different positions across the three systems. Comparative studies have documented both convergence and substantial divergence among global rankings (Moed, 2017; Moskovkin et al., 2022; Viana et al., 2024), while recent longitudinal evidence shows that agreement also depends on institutional coverage, ranking depth, and the metric used to define similarity (Kuldeyev et al., 2026).

This disagreement reflects a broader methodological controversy surrounding composite university rankings. League tables imply that heterogeneous dimensions of university performance can be combined into a meaningful common hierarchy, yet the resulting order is conditional on numerous analytical choices. Transformations and variable selection can materially affect composite outcomes (Williams and de Rassenfosse, 2016), nominal indicator weights do not necessarily correspond to their effective influence on final positions (Soh, 2013), and institutional ranks can change under alternative weighting assumptions (Pinar et al., 2019). From a responsible research assessment perspective, concerns have also been raised about construct validity, reputation measures, disciplinary and linguistic biases, data quality, uncertainty, reproducibility, and the reduction of multidimensional institutional missions to a single score or rank (Gadd et al., 2021; Latifis et al., 2026). The central controversy is therefore not simply which ranking is most accurate, but whether any single ranking architecture can adequately represent a complex and multidimensional institution.

Rank aggregation offers one response to this problem by combining several rankings into a consensus ordering rather than privileging one league table. A growing literature has explored voting-based, algorithmic, multicriteria, preference-based, and other aggregation approaches (Khodabakhshi and Aryavash, 2015; Zhang et al., 2017; Salimova et al., 2021; Manyoma-Velásquez, 2022; Feng et al., 2023). However, aggregation introduces its own methodological choices: different defensible aggregation rules can produce different consensus positions, while creating another composite ordering may conceal the very cross-system differences that motivated aggregation in the first place. This has encouraged complementary approaches that preserve cross-ranking profiles rather than forcing all institutions into a single linear hierarchy (Matlis et al., 2024).

Conceptually, these issues can be understood as a sequential evaluation problem. Heterogeneity in ranking architectures—including differences in indicators, weights, normalization, eligibility rules, and data treatment—can generate positional disagreement when the same institutions are evaluated across systems. Rank aggregation can summarize this disagreement into a consensus ordering, but such compression may entail information loss when materially different cross-ranking positions are reduced to a single rank. Where rankings are subsequently used for policy benchmarking, this information loss becomes especially consequential because fixed thresholds, such as Top-100 or Top-200 status, may yield different institutional classifications across ranking systems. The analytical framework of this study therefore links ranking-system heterogeneity, observed positional disagreement, consensus aggregation, preservation of cross-ranking differences, and policy-threshold interpretation as successive stages of the same evaluation problem.

A related line of research has sought to move beyond conventional global league tables through context-sensitive multicriteria evaluation. Pinto-Delacadena et al. (2024), for example, examined the criteria used by QS and SCImago in the Latin American context and applied TOPSIS to construct alternative university orderings based on multiple criteria and their assigned weights. Their approach emphasizes regional relevance and allows the incorporation of criteria, including economic and inclusion considerations, that may be underrepresented in conventional global rankings. The present study addresses a different but complementary problem. Rather than constructing a new context-specific ranking from indicator-level data, it evaluates the consistency of the published outputs of QS, THE, and ARWU, examines the robustness of alternative rank-aggregation procedures, and preserves disagreement among ranking systems through cross-ranking profile and Pareto assessment. Thus, whereas indicator-level multicriteria approaches can redefine the criteria and weights used to evaluate universities, the present framework is designed primarily to monitor and interpret the robustness and policy implications of existing global ranking outputs.

These issues have particular relevance to Saudi Arabia, where international university rankings have become intertwined with higher education development and national competitiveness. Previous research has traced the international ranking trajectories of Saudi universities (Faiz and Al-Mutairi, 2015), proposed national approaches for combining multiple international rankings (Alduais, 2019), examined factors associated with the rapid rise of Saudi institutions in global rankings (Alhuthali and Sayed, 2022), applied multicriteria decision-making approaches to the evaluation of higher education quality standards in Saudi university colleges (Makki et al., 2023), and questioned the applicability of global ranking structures to universities in the wider Arab region (Altakhaineh and Zibin, 2021). This literature demonstrates growing Saudi visibility in international rankings while also cautioning against equating improvement in league-table position with equivalent improvement across all dimensions of institutional performance.

The issue has explicit policy significance under Saudi Vision 2030. The Human Capability Development Program (HCDP) includes improving the ranking of educational institutions among its objectives and establishes targets for increasing the number of Saudi universities among the global top 100 and top 200. Importantly, the policy framework identifies QS, Times Higher Education, and Shanghai Ranking as the international systems used to measure this objective (Human Capability Development Program, 2021). This creates an unusual evaluation problem: national progress is being considered through three prominent ranking systems that do not necessarily define or measure university excellence in the same way. A university may therefore satisfy a policy-relevant threshold in one system while occupying a substantially different position in another. For policymakers and institutional leaders, the relevant question is consequently not only *where Saudi universities rank*, but also *how consistently and robustly their international standing is represented across the ranking systems used for policy benchmarking*.

Despite extensive international research on ranking methodologies and a growing Saudi literature on ranking performance, these strands have not yet been fully integrated. International studies have compared QS, THE, and ARWU and developed increasingly sophisticated approaches to cross-ranking analysis (Moskovkin et al., 2022; Kuldeyev et al., 2026), while Saudi studies have examined historical trajectories, ranking gains, and multi-ranking evaluation (Faiz and Al-Mutairi, 2015; Alduais, 2019; Alhuthali and Sayed, 2022). However, to our knowledge, previous Saudi research has not combined a year-aligned longitudinal comparison of the three systems explicitly referenced in national policy with alternative rank-aggregation procedures, multidimensional representation, and systematic robustness analysis. This distinction is important because disagreement between rankings may itself contain policy-relevant information that is lost when attention is restricted either to a single league table or to a single aggregated score.

Accordingly, this study examines the positions of Saudi universities in QS, THE, and ARWU over the aligned 2022–2025 period. It has four objectives: to determine the extent and temporal stability of cross-ranking agreement; to examine the consensus ordering produced by alternative rank-aggregation procedures; to identify institutional patterns that remain visible when the three rankings are retained as separate dimensions; and to test the robustness of these assessments to alternative aggregation procedures, weighting schemes, and rank-band treatments. The study thereby distinguishes three related but conceptually different questions: whether ranking systems agree, whether their results can be combined robustly, and what information is lost when disagreement is compressed into a single ordering.

Rather than seeking to construct another definitive league table, the study uses disagreement and sensitivity as evidence about the limits of ranking-based evaluation. The analysis is designed to show whether conclusions about Saudi universities remain stable across QS, THE, and ARWU and whether consensus rankings conceal substantively different institutional profiles. In this way, the study moves beyond the question of which Saudi university ranks highest toward a more policy-relevant assessment of how consistently, robustly, and multidimensionally international standing can be evaluated. The resulting framework is intended primarily as a monitoring and interpretive tool, enabling policymakers and institutional leaders to identify whether conclusions are stable across ranking systems, distinguish robust consensus from system-sensitive results, and interpret ranking-based policy thresholds more transparently. It is not intended to prescribe indicator-level institutional improvement strategies, which would require a different analytical design based on the underlying component indicators and scores of the ranking systems.

## 2 Materials and methods

### 2.1 Study design and analytical framework

This study employed a longitudinal, non-interventional quantitative design based exclusively on publicly available institutional-level data from three global university ranking systems: the QS World University Rankings (QS), the Times Higher Education World University Rankings (THE), and the Academic Ranking of World Universities (ARWU). The unit of observation was the university-ranking-year record. The primary analytical period was 2022–2025, providing four consecutive editions for which the three ranking systems could be aligned.

The three systems were selected for both methodological and policy reasons. They represent distinct approaches to the measurement of university performance and are among the most widely used international rankings (Moed, 2017; Latifis et al., 2026). More importantly for the national case examined here, the Human Capability Development Program (HCDP) under Saudi Vision 2030 explicitly identifies QS, Times Higher Education, and Shanghai Ranking in connection with its indicators for increasing the number of Saudi universities among the global top 100 and top 200.

The analytical framework comprised four complementary components: (1) cross-ranking coverage and agreement analysis; (2) longitudinal assessment of institutional rank variability; (3) consensus rank aggregation; and (4) cross-ranking profile and Pareto assessment supplemented by robustness and sensitivity analysis. Conceptually, these components were organized as a sequence linking heterogeneity in ranking-system design to observed positional disagreement, the construction of consensus through aggregation, the potential loss of information when disagreement is compressed into a single ordering, and uncertainty in policy-threshold interpretation. This structure allowed the analysis to distinguish between disagreement originating in the ranking systems themselves and sensitivity introduced when heterogeneous ranking outputs are combined or used for policy benchmarking.

### 2.2 Data sources and study period

Ranking data were collected from the official annual results published by QS, THE, and ShanghaiRanking for the 2022, 2023, 2024, and 2025 editions (QS Quacquarelli Symonds, 2023). Only information published by the ranking providers was used as primary ranking data. For each university-ranking-year record, the database retained the ranking provider, edition year, institution name as originally published, standardized institution identifier, published rank or rank band, and ranking status.

The official methodology documentation associated with the ranking systems was also examined to identify differences in eligibility criteria, indicators, weighting, normalization, data treatment, and publication practices (QS Quacquarelli Symonds, 2025; ShanghaiRanking Consultancy, 2024; Times Higher Education, 2023, 2024). These methodological differences were not treated as nuisance variation to be removed from the data; rather, they formed part of the interpretive context for cross-ranking disagreement.

Particular attention was paid to longitudinal comparability. Ranking editions were treated as annual measurement frameworks rather than repeated applications of an invariant instrument. Both QS and THE introduced substantial methodological revisions in their 2024 editions. QS implemented a major methodological enhancement that introduced three new indicators-Sustainability, Employment Outcomes, and International Research Network-alongside changes to the weighting structure of the ranking. THE likewise adopted a substantially revised methodology beginning with the 2024 edition, expanding from 13 performance indicators organized around five broad areas in the preceding editions to 18 indicators grouped into five pillars. The 2025 THE methodology retained the 18-indicator framework across Teaching, Research Environment, Research Quality, International Outlook, and Industry. Accordingly, changes in institutional positions across the 2023–2024 boundary were not interpreted automatically as changes in underlying institutional performance. Where informative, agreement patterns were additionally examined descriptively for 2022–2023 and 2024–2025 to assess whether the principal findings were sensitive to these methodological discontinuities. No causal effect of the methodology revisions was inferred.

### 2.3 . Institutional identification and data harmonization

A master institutional register was constructed to harmonize university identities across ranking systems and years. Each institution was assigned a standardized identifier while its original published name was retained for auditability. Variations involving capitalization, punctuation, abbreviations, transliteration, and minor naming differences were reconciled conservatively. Potential cases involving institutional renaming, restructuring, or merger were reviewed individually rather than resolved through unrestricted fuzzy matching.

The harmonized dataset was maintained in long format, with one record per university-ranking-year observation, and transformed into year-specific wide tables when pairwise or three-system comparisons were required. Institutional absence was distinguished from an observed low rank. A university not appearing in a published ranking was therefore recorded as not published rather than assigned an artificial bottom position.

THE “Reporter” institutions were treated as a separate status. According to THE methodology, Reporter institutions submit data but do not meet the eligibility criteria required to receive a World University Ranking position. They were consequently excluded from numerical rank comparisons and were never assigned a bottom rank. This distinction prevents participation in the ranking process from being incorrectly interpreted as an observed ranked position.

### 2.4 Treatment of exact ranks, rank bands, and missing observations

Published ranks were retained in their original form. Exact ranks were recorded as single values, whereas bounded rank bands were represented by their lower and upper limits. For procedures requiring a single numerical representation of a bounded band [*L*_*i*_, *U*_*i*_], the primary specification used its midpoint:


$$\begin{array}{l} m_{i}=\frac{L_{i}+U_{i}}{2}. \end{array}$$

The midpoint was treated solely as an operational representation of an interval and not as an estimate of the university's unknown exact rank. Robustness analyses therefore repeated applicable calculations using the lower and upper endpoints of each band.

Open-ended bands, such as THE's “1201+,” were not assigned an arbitrary midpoint because their upper boundary is undefined. Such observations were retained for coverage analysis but excluded from calculations requiring a finite numerical rank unless the analysis could be performed directly from ordinal or categorical information without imposing an artificial upper bound.

Missing ranks were not imputed. Pairwise comparisons between two ranking systems were based on universities jointly ranked by those systems in the same edition year. Analyses requiring simultaneous information from QS, THE, and ARWU were conducted on the complete three-system intersection for the relevant year. The number of eligible observations was reported with each analysis so that variation in institutional coverage was not conflated with variation in rank agreement.

### 2.5 Cross-ranking coverage and agreement

Coverage similarity and positional agreement were evaluated separately because two ranking systems may include many of the same institutions while ordering those institutions differently (Kuldeyev et al., 2026). For each year and each pair of ranking systems-QS-THE, QS-ARWU, and THE-ARWU-the number of Saudi universities jointly ranked by the two systems was first determined.

Institutional coverage overlap was quantified using the Jaccard coefficient:


$$\begin{array}{l} J\left(A,B\right)=\frac{|A\cap B|}{|A\cup B|}, \end{array}$$

where *A* and *B* denote the sets of Saudi universities with published ranking positions in two systems during the same year. Values range from 0 to 1, with *J* = 1 indicating identical institutional coverage and *J* = 0 indicating no overlap. Institutions classified by THE solely as *Reporters* were not included in the ranked institutional set because they did not receive a published World University Ranking position.

Positional agreement was assessed only among universities jointly ranked by the relevant pair of systems in the same year. For bounded rank bands, the midpoint representation described in Section 2.4 was used in the primary numerical analysis. Identical published ranks or rank bands were retained as ties. Open-ended bands without a finite upper boundary were excluded from correlations requiring a finite numerical rank rather than being assigned an arbitrary value. Consequently, the number of observations used for positional agreement could be smaller than the corresponding institutional coverage overlap.

Two complementary non-parametric measures were used to quantify positional agreement: Spearman's rank correlation coefficient (ρ) and Kendall's rank correlation coefficient (τ). Spearman's ρassesses the monotonic correspondence between the positions assigned by two ranking systems, whereas Kendall's τevaluates concordance in pairwise ordering and accommodates tied positions arising from published rank bands. Using both measures reduced reliance on a single definition of rank agreement.

The analysis emphasized the magnitude, direction, and consistency of the correlation coefficients across ranking pairs and years rather than relying primarily on null-hypothesis significance testing. This approach was appropriate because the analysis covered the available set of Saudi universities jointly published by the selected ranking systems in each edition rather than a probability sample drawn for inferential generalization. Cross-ranking agreement was therefore interpreted descriptively and comparatively, with particular attention to whether patterns persisted across years and ranking pairs. The coefficients should nevertheless be interpreted cautiously, particularly for ranking pairs and years with small numbers of jointly ranked institutions and with tied positions arising from published rank bands. Accordingly, the reported Spearman and Kendall coefficients are treated as descriptive measures of positional agreement within the observed year-specific common sets rather than as precise estimates of a broader relationship between the ranking systems.

### 2.6 Longitudinal institutional rank variability

Longitudinal patterns were examined for Saudi universities appearing repeatedly within the same ranking system during the 2022–2025 period. The analysis tracked each institution's published exact rank or rank band across successive annual editions of QS, THE, and ARWU, with the aim of identifying whether institutional trajectories were broadly consistent across ranking systems or displayed persistent cross-system divergence.

Because published global ranks are relative positions within ranking populations that can change in size and composition from year to year, longitudinal movements were interpreted descriptively rather than as direct measures of changes in institutional quality. A change in published position may reflect changes in the institution itself, the performance or inclusion of competing universities, underlying data, indicator construction, normalization procedures, or ranking methodology. Accordingly, the analysis focused on the direction, magnitude, and cross-system consistency of observed positional trajectories rather than treating annual rank changes as interval-scale measures of institutional improvement or decline.

Particular caution was applied around the 2023–2024 transition because both QS and THE introduced substantial methodological revisions for their 2024 editions. Rank movements across this boundary were therefore not attributed automatically to changes in institutional performance, and no causal effect of the methodological revisions was inferred.

The longitudinal analysis was conducted across all institutions with repeated observations within the relevant ranking system during 2022–2025. Institutional trajectories were first examined across the eligible longitudinal records, with attention to the direction and magnitude of positional change and to cross-system consistency where institutions were represented in more than one ranking. Three institutions—KAU, KSU, and KFUPM—were subsequently selected for detailed presentation because they exhibited particularly clear, persistent, and substantively large patterns of cross-ranking divergence over the study period. These cases were therefore illustrative rather than constituting the full longitudinal sample. They were interpreted alongside the consensus and cross-ranking profile analyses to distinguish broadly shared ranking signals from system-specific patterns. Given the substantial methodological revisions introduced by QS and THE for their 2024 editions, interpretation also distinguished, where relevant, between patterns observed in 2022–2023 and those observed in 2024–2025; this descriptive separation was not treated as a causal pre/post analysis.

### 2.7 Rank-aggregation procedures

Consensus rankings were constructed separately for each year using only universities that were jointly ranked by QS, THE, and ARWU. Before aggregation, the published global ranks, including bounded rank-band representations, were converted into within-year ordinal positions among institutions in the common analytical set. Universities sharing the same published rank or rank band were assigned tied ordinal positions using average ranks; alphabetical ordering within published bands was not treated as a substantive ranking distinction.

Because no single aggregation rule can be regarded as uniquely authoritative, three structurally distinct procedures were selected to represent different conceptions of consensus: Borda positional aggregation captures cumulative positional performance across all ranking systems; median-rank aggregation captures the central ordinal position and reduces the influence of one strongly divergent ranking; and Copeland aggregation captures pairwise majority preference across the ranking systems. Comparing these procedures therefore tests whether the resulting consensus depends on an additive, central-position, or majority-based definition of agreement. Agreement across the three methods was interpreted as evidence of structural stability in the consensus ordering, whereas disagreement was treated as evidence of sensitivity to the aggregation rule rather than as an error to be eliminated (Feng et al., 2023).

Under the Borda procedure, the ordinal position of university *i* in ranking system *s*, denoted *r*_*is*_, was converted into an inverse positional score. For a common set containing *n*universities, the Borda score was calculated as


$$\begin{array}{l} B_{i}=\overset{3}{\underset{s=1}{\sum }}\left(n-r_{is}+1\right), \end{array}$$

where higher values indicate stronger consensus performance. Average tied positions were retained in the score calculation so that institutions assigned to the same published rank band were not artificially separated.

The median-rank procedure summarized the central ordinal position of each university across the three systems:


$$\begin{array}{l} M_{i}=\text{median}\left(r_{i,\;QS},\;r_{i,\;THE},\;r_{i,\;ARWU}\right). \end{array}$$

Lower median values indicate stronger consensus standing. Because the median is relatively insensitive to one strongly divergent component rank, it provided a robust complement to the additive Borda procedure.

Copeland aggregation was used as a third, structurally distinct method (Saari and Merlin, 1996). For every pair of universities *i* and *j*, their relative positions were compared independently across QS, THE, and ARWU. University *i* was considered to defeat university *j* when a majority of the three ranking systems placed *i* in a better ordinal position than *j*. A pairwise tie was recorded when neither university obtained a majority advantage, including cases in which the component comparisons resulted in an overall tie. The Copeland score for university *i* was defined as


$$\begin{array}{l} C_{i}=W_{i}-L_{i}, \end{array}$$

where *W*_*i*_ denotes the number of pairwise victories and *L*_*i*_ denotes the number of pairwise defeats. Universities were ordered from the highest to the lowest Copeland score, with equal scores retained as ties. Unlike Borda aggregation, which summarizes cumulative ordinal positions across the three ranking systems, Copeland aggregation derives the consensus ordering from pairwise majority relations.

Arithmetic mean rank was not treated as a separate aggregation method. For an identical complete set of universities under equal system weights, mean rank and Borda aggregation are monotonic transformations of the same positional information and therefore generate the same ordering. Treating them as independent procedures would consequently overstate methodological diversity.

Agreement among the three consensus solutions was evaluated using Spearman and Kendall rank correlations and by examining absolute changes in institutional consensus position. Universities whose placement changed materially across Borda, median-rank, and Copeland procedures were identified as aggregation-sensitive cases. This comparison was used to determine whether the combined ordering was robust to the choice of aggregation rule rather than assuming that any single consensus ranking represented a uniquely correct institutional hierarchy.

### 2.8 Cross-ranking profile and Pareto assessment

Rank aggregation was complemented by a cross-ranking profile analysis in which the outputs of QS, THE, and ARWU were retained separately rather than collapsed immediately into a single consensus position. The three ranking systems were treated as distinct composite evaluation outputs, not as independent dimensions of underlying university performance such as teaching, research, or internationalization. The purpose of this representation was therefore to preserve disagreement among the ranking systems and to examine whether institutional comparisons remained consistent across their published outputs.

For descriptive comparability within each year-specific common set, ordinal positions were normalized as


$$\begin{array}{l} P_{is}=1-\frac{\left(r_{is}-1\right)}{\left(N_{s}-1\right)}, \end{array}$$

where r_is is the ordinal position of university i in ranking system s, and N_s is the number of universities in the relevant comparison set. Values closer to 1 indicate stronger relative standing within that analytical set. These normalized values were interpreted solely as within-sample relative positions in the respective ranking systems, not as globally comparable university performance scores or direct measures of underlying institutional performance dimensions.

Each institution was consequently represented by a cross-ranking profile:


$$\begin{array}{l} {\text{P}}_{i}=\left(P_{i,QS},P_{i,THE},P_{i,ARWU}\right) \end{array}$$

Pareto dominance was used to identify institutions whose cross-ranking profiles were unambiguously stronger within the year-specific common analytical set. University i was defined as dominating university j if it occupied an equal or stronger normalized position in all three ranking systems and a strictly stronger position in at least one. Institutions for which neither dominated the other were retained as incomparable rather than forced into an artificial linear ordering. The resulting Pareto frontier therefore represents non-dominated institutions only among the universities jointly ranked by QS, THE, and ARWU in the relevant year; it should not be interpreted as identifying the non-dominated universities in the Saudi higher education sector as a whole. This partial-order perspective complements the consensus ranking by preserving cross-system trade-offs that would otherwise be obscured by a single aggregated ordering (Latifis et al., 2026; Matlis et al., 2024).

### 2.9 Robustness and sensitivity analyses

Robustness and sensitivity analyses were conducted to determine whether the principal consensus findings depended on reasonable alternative analytical assumptions. Three sources of sensitivity were examined: the choice of rank-aggregation procedure, the relative weighting assigned to the three ranking systems, and the numerical treatment of bounded rank intervals.

Aggregation-method sensitivity was assessed by comparing the consensus orderings produced by Borda positional aggregation, median-rank aggregation, and Copeland pairwise-majority aggregation, as described in Section 2.7. Agreement among the resulting consensus rankings was evaluated using Spearman's and Kendall's rank correlations and by examining absolute changes in institutional positions. This comparison assessed whether the principal consensus structure persisted across aggregation procedures representing different conceptions of consensus: cumulative positional performance, central ordinal position, and pairwise majority preference.

The baseline Borda aggregation assigned equal weight to QS, THE, and ARWU. Weight sensitivity was examined in two complementary ways. First, three interpretable asymmetric scenarios were evaluated in which one ranking system received a weight of 0.50 and each of the remaining systems received a weight of 0.25:


$$\begin{array}{l} \left(W_{QS},W_{THE},W_{ARWU}\right)\in \left\{\left(0.50,0.25,0.25\right),\left(0.25,0.50,0.25\right),\left(0.25,0.25,0.50\right)\right\}. \end{array}$$

For these scenarios, the weighted Borda score for university (i) was calculated as


$$\begin{array}{l} B_{i}^{\left(w\right)}=\overset{3}{\underset{s=1}{\sum }}W_{s}\left(n-r_{is}+1\right), \end{array}$$

where (w_s) denotes the weight assigned to ranking system (s), (r_{is}) is the within-year ordinal position of university (i) in that system, and (n) is the number of universities in the year-specific three-system common set. The asymmetric scenarios were retained as readily interpretable sensitivity tests rather than as substantive claims about the appropriate relative importance of QS, THE, or ARWU.

Second, the discrete weighting scenarios were complemented by a continuous weight-space analysis. The admissible weight space was defined by


$$\begin{array}{l} W_{QS}+W_{THE}+W_{ARWU}=1,\;W_{QS},W_{THE},W_{ARWU}\geq 0. \end{array}$$

Weighted Borda scores were evaluated across this simplex to identify the combinations of ranking-system weights under which each university occupied the leading consensus position. This analysis was used to distinguish regions of leadership stability from transition boundaries at which the identity of the leading institution changed, with ties retained where two or more institutions obtained equal weighted Borda scores. The continuous analysis therefore extended the discrete sensitivity scenarios by evaluating leadership stability across the full range of admissible relative weights rather than only at a small number of pre-specified weighting combinations.

Rank-band sensitivity was evaluated through a deterministic endpoint analysis in which the applicable Borda calculations were repeated using the lower endpoint, midpoint, and upper endpoint of each bounded published rank interval before deriving the within-year ordinal positions. Open-ended intervals were not assigned artificial upper endpoints. The resulting consensus orderings were compared with the midpoint-based baseline to assess whether these alternative deterministic representations of bounded rank intervals changed the observed consensus ordering. This procedure was treated as a limited sensitivity check rather than as a complete representation of uncertainty within rank bands, because converting interval endpoints into ordinal positions can preserve relative ordering even when the exact positions of institutions within the published bands remain unknown.

Robustness was summarized using correlations among alternative consensus rankings and absolute changes in institutional consensus positions. For the discrete weighting scenarios, particular attention was given to whether universities remained within one or two positions of their equal-weight Borda baseline, and the maximum absolute positional change was recorded for each year. The continuous weight-space analysis additionally assessed the stability of the leading institution across the admissible weighting simplex and identified the weight combinations associated with changes in leadership. For the rank-band analysis, the lower- and upper-endpoint specifications were compared with the midpoint baseline to identify whether these deterministic representations changed the consensus ordering. An unchanged ordering was interpreted only as stability under the tested endpoint specifications, not as evidence that uncertainty within published rank bands had been fully resolved. Cases exhibiting comparatively large or substantively important changes—particularly changes affecting the leading consensus position—were examined individually rather than being obscured by overall measures of rank stability.

### 2.10 Policy interpretation under Saudi vision 2030

The HCDP policy indicators were analyzed separately from the consensus-ranking exercise. The official top-100 and top-200 thresholds were therefore not treated as weights or optimization criteria in the aggregation algorithms.

For each system and year, an institution was classified as meeting a policy threshold when its exact rank or the entire published rank band fell within that threshold. A bounded rank interval spanning a policy cutoff would be classified as indeterminate rather than assigned to either side of the threshold using its midpoint. Institutions with rank bands beginning below the relevant threshold-for example, 201–250 in relation to a top-200 criterion-were classified as outside that threshold. Reporter and unpublished institutions were not assumed to fail the threshold on the basis of an invented rank.

The policy analysis examined whether conclusions concerning the number and identity of Saudi universities satisfying the top-100 and top-200 criteria were consistent across QS, THE, and ARWU. This component was interpretive and descriptive; the study did not attempt to estimate a causal effect of Vision 2030 or the HCDP on university ranking performance. Rather, it was designed to support transparent monitoring and interpretation of ranking-based policy outcomes by identifying whether threshold achievement was consistent or system-dependent across the three policy-referenced rankings. It was not intended to identify indicator-level strategies for improving institutional rank, which would require a different analytical design and underlying component-level data.

### 2.11 Statistical analysis and reproducibility

Data harmonization, statistical analysis, rank aggregation, cross-ranking profile assessment, and sensitivity analyses were implemented in Python. Custom analytical procedures were used for institutional harmonization, coverage and overlap calculations, rank-band transformations, Borda, median-rank, and Copeland aggregation, cross-ranking normalization, Pareto-dominance assessment, discrete and continuous weight-sensitivity analyses, policy-threshold classification, and rank-band robustness analyses. All reported numerical results were generated from the harmonized analytical dataset using these procedures and were checked for consistency with the underlying ranking records.

Spearman's rank correlation coefficient and Kendall's rank correlation coefficient were used as descriptive measures of positional agreement. Given that the study analyzes the available population of Saudi universities jointly represented in the selected ranking systems rather than a probability sample drawn for inferential generalization, interpretation focused primarily on the magnitude, direction, and consistency of the coefficients across ranking pairs and years. Analytical sample sizes were reported alongside the corresponding agreement estimates.

Reproducibility was supported through preservation of the analytical code, institutional harmonization rules, rank-band treatment procedures, derived analytical tables, and documentation of the official source used for each ranking edition. Original ranking-provider data are not redistributed where provider terms or copyright restrictions prohibit such redistribution; in such cases, the reproducibility materials identify the corresponding official source and document the procedures required to reconstruct the analytical dataset. Details of data and code availability are provided in the Data Availability Statement.

### 2.12 Ethical considerations

The study uses publicly available institutional-level ranking information and policy and methodology documents. It involves no human participants, identifiable personal data, clinical information, animals, or experimental intervention. Accordingly, ethical approval and informed consent were not required.

### 2.13 Use of generative artificial intelligence

Generative artificial intelligence (GenAI) was used as a research-support tool during the development of this study, including assistance with methodological structuring, analytical planning, computer-code development, and language refinement. GenAI was not used as a source of university ranking data and was not permitted to generate or substitute empirical observations. All ranking records used in the analysis were obtained from the identified official ranking-provider sources.

All data harmonization decisions, analytical procedures, computer code, numerical outputs, references, and interpretations were reviewed and verified by the authors against the original sources and reproducible analytical outputs. GenAI did not autonomously determine the study findings or conclusions. The authors retain full responsibility for the integrity, accuracy, and interpretation of the work.

## 3 Results

### 3.1 Institutional coverage across ranking systems

The three ranking systems differed substantially in their coverage of Saudi universities over the 2022–2025 period (Table 1). QS increased its Saudi coverage from 14 ranked institutions in 2022 to 20 in 2025, while THE increased from 15 to 26 ranked institutions. ARWU remained more selective, publishing seven Saudi institutions in 2022, 12 in both 2023 and 2024, and 13 in 2025.

**Table 1:** Institutional coverage and pairwise overlap of Saudi universities, 2022–2025.

Year	QS ranked	THE ranked	ARWU ranked	QS–THE overlap	QS–ARWU overlap	THE–ARWU overlap	Three-system overlap
2022	14	15	7	8	4	6	4
2023	16	21	12	13	9	11	9
2024	16	22	12	13	8	11	8
2025	20	26	13	19	9	12	9

The number of institutions that could be compared directly across all three systems consequently remained considerably smaller than the coverage of QS or THE individually. The three-system intersection comprised four universities in 2022, nine in 2023, eight in 2024, and nine in 2025. Pairwise overlap increased most strongly between QS and THE, reaching 19 shared universities in 2025.

Coverage similarity, measured using the Jaccard coefficient, confirmed that the rankings did not contain identical institutional populations. QS-THE overlap increased from *J* = 0.381 in 2022 to *J* = 0.704 in 2025. In contrast, QS–ARWU overlap remained lower, ranging from 0.235 to 0.474, while THE–ARWU ranged from 0.375 to 0.500. Thus, increased Saudi representation in the individual rankings did not eliminate differences in institutional coverage.

### 3.2 Cross-ranking positional agreement

Pairwise rank correlations showed that the three systems were positively associated but not interchangeable (Table 2). QS and THE displayed the strongest and most persistent agreement overall. Their Spearman correlation was exceptionally high in 2022 (ρ = 0.982) and 2025 (ρ = 0.932), although it was more moderate in 2023 (ρ = 0.726) and 2024 (ρ = 0.626). Kendall coefficients showed the same pattern.

**Table 2:** Pairwise coverage and positional agreement among QS, THE, and ARWU.

Year	Ranking pair	Shared n	Numerical n	Jaccard	Spearman ρ	Kendall τ
2022	QS–THE	8	7	0.381	0.982	0.951
	QS–ARWU	4	4	0.235	0.632	0.548
	THE–ARWU	6	5	0.375	0.816	0.667
2023	QS–THE	13	13	0.542	0.726	0.634
	QS–ARWU	9	9	0.474	0.609	0.469
	THE–ARWU	11	11	0.500	0.480	0.372
2024	QS–THE	13	13	0.520	0.626	0.521
	QS–ARWU	8	8	0.400	0.537	0.416
	THE–ARWU	11	11	0.478	0.643	0.546
2025	QS–THE	19	19	0.704	0.932	0.816
	QS–ARWU	9	9	0.375	0.437	0.286
	THE–ARWU	12	12	0.444	0.594	0.479

“Shared *n*” denotes institutional overlap used for coverage analysis. “Numerical *n*” denotes observations with finite numerical rank representations available for positional correlation. Open-ended rank bands were not assigned artificial upper bounds.

ARWU generally exhibited weaker correspondence with the other systems. QS-ARWU Spearman coefficients ranged from 0.437 in 2025 to 0.632 in 2022, whereas THE-ARWU correlations ranged from 0.480 in 2023 to 0.816 in 2022. The 2022 QS-THE and THE-ARWU numerical samples were one institution smaller than their coverage overlaps because THE's open-ended 1,201+ category could not be assigned a finite midpoint.

The year-specific pattern is particularly informative. In 2025, QS and THE were highly concordant (ρ = 0.932, τ = 0.816), whereas QS–ARWU agreement was much weaker (ρ = 0.437, τ = 0.286). Thus, even in the year with the greatest QS-THE coverage overlap, convergence between the three systems as a whole did not occur.

### 3.3 Longitudinal variability and institutional divergence

The longitudinal review of institutions with repeated observations showed that published trajectories were not necessarily consistent across ranking systems. To illustrate the clearest cases of persistent and substantively large cross-ranking divergence, three institutions—King Saud University (KSU), King Fahd University of Petroleum and Minerals (KFUPM), and King Abdulaziz University (KAU)—were examined in greater detail. These cases were selected as illustrations of the broader longitudinal comparison rather than as the complete longitudinal sample.

KSU displayed a pronounced cross-ranking divergence in the post-2024 methodology period. In 2024, it was positioned at 203 in QS and 401–500 in THE, whereas ARWU ranked it 90th globally. In 2025, its corresponding positions were 200 in QS, 251–300 in THE, and 101–150 in ARWU. KSU therefore maintained a substantially stronger relative position in ARWU than in QS or THE across both 2024 and 2025.

KFUPM exhibited a contrasting pattern. In 2024, it was ranked 180 in QS and 201–250 in THE but remained in the 701–800 band in ARWU. In 2025, its QS and THE positions improved to 101 and 176, respectively, while its ARWU position remained in the 701–800 band. Thus, the substantial separation between its QS/THE and ARWU positions persisted across the two editions following the major QS and THE methodological revisions.

KAU displayed a different longitudinal pattern. Its ARWU position moved successively from 101–150 in 2022 to 151–200 in 2023, 201–300 in 2024, and 501–600 in 2025. Its QS positions over the same period were 109, 106, 143, and 149, respectively. The divergence was therefore visible not only across ranking systems but also across the 2023–2024 methodological transition. Because that transition coincided with substantial revisions to the QS and THE methodologies, the observed movements cannot be attributed solely to changes in underlying institutional performance.

Taken together, these illustrative cases show that cross-ranking disagreement was not confined to marginal differences among similarly positioned universities. In several instances, the same institution occupied substantially different segments of the global ranking distribution, and some of these differences persisted across successive editions. The longitudinal evidence is therefore interpreted descriptively: it demonstrates persistent cross-ranking divergence in selected illustrative cases but does not establish that ranking-methodology changes caused the observed institutional movements.

### 3.4 Consensus rank aggregation

Consensus rankings were calculated separately for each year using universities jointly ranked by all three systems. Table 3 compares equal-weight Borda aggregation with Median Rank and Copeland pairwise-majority aggregation.

**Table 3:** Alternative consensus rankings for universities jointly ranked by QS, THE, and ARWU.

Year	University	QS position	THE position	ARWU position	Borda	Median	Copeland
2022	KAU	1	1	1.5	1	1	1
	KSU	3	2.5	1.5	2	2.5	2.5
	KFUPM	2	2.5	3	3	2.5	2.5
	KKU	4	4	4	4	4	4
2023	KAU	1	1	2	1	1	1
	KSU	3	3	1	2	3	3
	KFUPM	2	2	4.5	3	2	2
	UQU	4	4.5	7	4	4	4
	PNU	6	7	3	5	6	5
	KKU	6	7	4.5	6	6	7
	Jouf	6	4.5	9	7	6	6
	KFU	8.5	7	7	8	8	8
	Qassim	8.5	9	7	9	9	9
2024	KAU	1	2	2	1	1.5	1
	KSU	3	3	1	2	3	3
	KFUPM	2	1	6	3	1.5	2
	PNU	5	5.5	3	4	5	4
	KKU	6	4	4	5	4	5
	UQU	4	5.5	6	6	6	6
	Qassim	8	7.5	6	7	7.5	7.5
	KFU	7	7.5	8	8	7.5	7.5
2025	KSU	3	2	1	1	2	2
	KFUPM	1	1	5	2	1	1
	KAU	2	4	4	3	4	4
	KKU	5	3	3	4	3	3
	PNU	6	6	2	5	6	6
	UQU	4	5	8.5	6	5	5
	KFU	7	8	6.5	7	7	7
	Qassim	8	8	6.5	8	8	8
	IMSIU	9	8	8.5	9	9	9

Component positions are ordinal positions within the year-specific three-system common set, not published global ranks. Fractional positions indicate ties.

The alternative aggregation methods were strongly concordant. Borda-Median Spearman correlations ranged from 0.928 to 0.950 across the four years, while Borda-Copeland correlations ranged from 0.949 to 0.970. Median and Copeland were identical in rank order in 2022 and 2025 (ρ = 1.000) and remained highly concordant in 2023 (ρ = 0.983) and 2024 (ρ = 0.970).

Nevertheless, the leading position was not completely method-independent. KAU was the consensus leader under all three procedures in 2022 and 2023 and remained first under Borda and Copeland in 2024. In 2025, however, Borda placed KSU first and KFUPM second, whereas Median and Copeland placed KFUPM first and KSU second. This difference arose from the pronounced ARWU-QS/THE divergence: KSU ranked first within the ARWU common set but third and second in QS and THE, respectively, whereas KFUPM ranked first in both QS and THE but fifth in ARWU.

Thus, aggregation produced a generally stable ordering but did not eliminate the substantive trade-offs among the three component systems.

### 3.5 Multidimensional assessment and Pareto frontiers

The Pareto analysis further demonstrated why a consensus ranking should not be interpreted as a complete representation of institutional standing. In 2022, KAU was the sole institution on the Pareto frontier: no other university matched or exceeded its relative position across all three systems (Table 4).

**Table 4:** Pareto frontier and dominance structure.

Year	Pareto-frontier institutions	Number on frontier	Most extensive dominance relation
2022	KAU	1	KAU dominated all 3 other institutions
2023	KAU; KSU	2	KAU dominated 7; KSU dominated 6
2024	KAU; KSU; KFUPM	3	KAU and KSU each dominated 5
2025	KAU; KSU; KFUPM	3	KSU dominated 6

In 2023, the frontier expanded to KAU and KSU. KAU had the strongest positions in QS and THE, whereas KSU held the strongest ARWU position. Neither therefore dominated the other across all three dimensions.

The frontier expanded again in 2024 to KAU, KSU, and KFUPM and remained composed of these three universities in 2025. Their profiles were substantively different. In 2025, normalized within-sample positions for KSU were 0.750 in QS, 0.875 in THE, and 1.000 in ARWU; KFUPM scored 1.000, 1.000, and 0.500, respectively; and KAU scored 0.875, 0.625, and 0.625. None of the three therefore dominated either of the others.

The 2025 results illustrate the difference between scalar consensus and multidimensional evaluation particularly clearly. Although Borda ranked KSU first, this did not imply that KSU was uniformly superior to KFUPM or KAU. KFUPM was stronger in QS and THE, while KSU was stronger in ARWU. The Pareto representation therefore retained a trade-off that necessarily disappeared when the three dimensions were compressed into a single consensus position.

### 3.6 Robustness and sensitivity analysis

The equal-weight Borda consensus was first tested against three asymmetric weighting scenarios in which QS, THE, or ARWU received a weight of 0.50 and the remaining systems each received 0.25. Overall, the consensus rankings were relatively robust to these moderate weight changes (Table 5).

**Table 5:** Sensitivity of equal-weight Borda positions to alternative system weights.

Year	Main sensitivity finding	Maximum absolute position change
2022	KSU and KFUPM exchanged positions under QS-dominant weighting	1
2023	UQU moved from 4th to 6th under ARWU-dominant weighting	2
2024	Changes were limited to one position or tied intermediate positions	1
2025	KSU and KFUPM exchanged the leading position under QS-dominant weighting	1

Across all three asymmetric weighting scenarios, every institution remained within two positions of its equal-weight baseline. With the single exception of UQU in the 2023 ARWU-dominant scenario, every institutional position remained within one position of baseline. The upper and lower portions of the consensus rankings were generally more stable than several intermediate positions.

The discrete scenarios were complemented by a continuous analysis of the full admissible weight space, defined by (w_{QS}+w_{THE}+w_{ARWU}=1) and non-negative system weights. This analysis revealed substantial differences in leadership stability across years. In 2022, KAU remained the leading institution throughout the interior of the admissible weight space, with a boundary tie with KSU only at the extreme ARWU-only vertex. In 2023, KAU occupied the leading position across approximately 88.9% of the admissible weight space, whereas KSU became the leader only in the strongly ARWU-dominant region where (w_{ARWU}>2/3).

Leadership was more weight-sensitive in 2024. KAU led across approximately 73.2% of the admissible weight space, compared with approximately 16.7% for KSU and 10.0% for KFUPM. The three institutions therefore occupied distinct leadership regions, reflecting their contrasting positions across the component ranking systems.

The greatest leadership sensitivity occurred in 2025, when only KSU and KFUPM occupied substantive leadership regions. KSU led across approximately 53.3% of the admissible weight space and KFUPM across approximately 46.7%. Their transition boundary was defined by


$$\begin{array}{c} 6W_{QS}+5W_{THE}=4. \end{array}$$

KSU occupied the leading position when


$$\begin{array}{c} 6W_{QS}+5W_{THE}<4, \end{array}$$

corresponding to combinations that placed relatively greater emphasis on ARWU, whereas KFUPM led when


$$\begin{array}{c} 6W_{QS}+5W_{THE}>4, \end{array}$$

corresponding to combinations that placed greater combined emphasis on QS and THE. At the boundary, the two institutions obtained equal weighted Borda scores. The equal-weight specification lies on the KSU side of this boundary, consistent with the baseline Borda result.

The continuous weight-space analysis therefore qualifies the apparent stability observed under the three discrete asymmetric scenarios. Leadership was highly stable in 2022 and remained stable across most admissible weights in 2023, but became progressively more weight-dependent in 2024 and especially in 2025. The 2025 result is particularly important because the identity of the consensus leader depends on a substantial trade-off between the ARWU advantage of KSU and the QS-THE advantage of KFUPM rather than on an isolated pre-specified weighting scenario.

Rank-band sensitivity produced a different pattern. Replacing bounded-band midpoints with their lower or upper endpoints produced no change in the equal-weight Borda ordering in any of the 4 years. The equal-weight Borda ordering was therefore stable under the lower-, midpoint-, and upper-endpoint representations tested. This result should be interpreted as stability under these deterministic specifications rather than as evidence that uncertainty within published rank bands was fully resolved.

### 3.7 Policy-relevant top-100 and top-200 thresholds

Application of the HCDP-relevant top-100 and top-200 thresholds produced markedly different conclusions depending on the ranking system (Table 6). No midpoint approximation was used for threshold classification.

**Table 6:** Saudi universities satisfying Top-100 and Top-200 thresholds, by ranking system.

Year	System	Top 100 *n*	Top 200 *n*	Universities within top 200
2022	QS	0	2	KAU; KFUPM
	THE	0	1	KAU
	ARWU	0	2	KAU; KSU
2023	QS	0	2	KAU; KFUPM
	THE	0	1	KAU
	ARWU	0	2	KSU; KAU
2024	QS	0	2	KAU; KFUPM
	THE	0	0	None
	ARWU	1	1	KSU
2025	QS	0	3	KFUPM; KAU; KSU
	THE	0	1	KFUPM
	ARWU	0	1	KSU

Top 200 includes institutions within the Top 100. Classification uses exact published ranks or bands lying wholly within the relevant threshold.

The sharpest disagreement occurred in 2024. ARWU ranked KSU 90th, making it the only Saudi institution in the Top 100 and Top 200 under that system. QS, by contrast, ranked KSU 203rd and therefore just outside the Top 200, while THE placed it in the 401–500 band. At the same time, QS placed KAU (143) and KFUPM (180) within its Top 200, whereas THE contained no Saudi university within its Top 200.

The disagreement persisted in 2025 but changed in form. QS identified three Saudi universities within the Top 200-KFUPM (101), KAU (149), and KSU (200). THE identified only KFUPM (176), whereas ARWU identified KSU through its 101–150 band. Thus, the same nominal policy threshold produced not only different counts but also different institutional identities across the three policy-referenced ranking systems.

### 3.8 Overall pattern of results

Taken together, the analyses yield four principal findings. First, Saudi institutional coverage expanded during 2022–2025, particularly in QS and THE, but substantial differences in coverage remained. Second, QS and THE generally exhibited stronger positional agreement than either system did with ARWU, although the magnitude of agreement varied by year. Third, consensus aggregation was relatively robust overall, but the identity of the leading university was sensitive when the component rankings strongly disagreed, most clearly for KSU and KFUPM in 2025. Fourth, multidimensional and policy-threshold analyses showed that consensus should not be interpreted as equivalence among the underlying rankings: KAU, KSU, and KFUPM could simultaneously occupy the Pareto frontier while presenting markedly different QS, THE, and ARWU profiles.

Accordingly, the empirical evidence does not support treating QS, THE, and ARWU as interchangeable measures of Saudi universities' international standing. At the same time, their disagreement exhibits identifiable system- and institution-specific patterns: a comparatively stable consensus can often be recovered, but that consensus conceals institution-specific trade-offs that remain important for benchmarking and for interpreting policy-relevant Top-100 and Top-200 thresholds.

## 4 Discussion

This study examined whether the international standing of Saudi universities can be represented consistently when three policy-relevant global ranking systems-QS, THE, and ARWU-are considered jointly rather than individually. The findings support the central premise of the study: the three systems capture related but non-equivalent dimensions of university performance. Their institutional coverage differed, their positional agreement varied substantially by ranking pair and year, and the same university could occupy markedly different locations across the three systems. At the same time, alternative rank-aggregation procedures produced broadly similar consensus structures, suggesting that a relatively stable combined ordering can often be recovered. However, the multidimensional and policy-threshold analyses showed that such consensus should not be mistaken for equivalence among the underlying rankings. The principal implication is therefore not that one system is superior to the others, but that institutional and policy conclusions become more defensible when disagreement, aggregation sensitivity, and multidimensional variation are treated as information rather than noise.

### 4.1 Cross-ranking agreement and the non-interchangeability of global rankings

The strongest empirical pattern was the comparatively high correspondence between QS and THE, particularly in 2022 and 2025, alongside weaker and less stable agreement between ARWU and the other systems. This pattern is consistent with previous comparative research showing that major global university rankings are positively associated but cannot be treated as interchangeable representations of institutional quality (Moed, 2017; Moskovkin et al., 2022; Viana et al., 2024). Kuldeyev et al. (2026) similarly demonstrated that the apparent similarity of QS, THE, and ARWU depends on the ranking pair, the depth of the comparison, and the metric used to assess similarity.

The Saudi results provide a particularly clear national illustration of this problem. In 2025, QS and THE exhibited very strong positional agreement, whereas the relationship between QS and ARWU was markedly weaker. This indicates that disagreement was not simply a consequence of different numbers of institutions being included. Even within a common set of universities, the systems could generate substantially different relative orderings.

A plausible methodological interpretation, rather than a directly tested causal explanation, is that these differences are related to the distinct measurement architectures of the ranking systems. QS and THE share several broad areas of emphasis related to research visibility, reputation, internationalization, and institutional environment, although they operationalize and weight these areas differently. ARWU, by contrast, places substantial emphasis on research-intensive indicators and internationally visible scientific achievement, including highly cited researchers, publications in *Nature* and *Science*, indexed research output, Nobel Prizes and Fields Medals, and per-capita academic performance. These methodological differences are consistent with the observed cross-ranking divergence. However, because the present study analyzes published ranks rather than indicator-level scores, it cannot establish which specific indicators, weights, or institutional characteristics caused the observed differences in institutional positions.

This interpretation is consistent with the broader literature on composite rankings. Williams and de Rassenfosse (2016) demonstrated that rankings are affected not only by nominal weights but also by indicator selection and data transformations, while Soh (2013) showed that the effective contribution of indicators can differ from their stated weights. Pinar et al. (2019) further demonstrated that ranking outcomes can be sensitive to alternative weighting assumptions, particularly outside the very top of global league tables. The present findings therefore support a more cautious interpretation of cross-ranking disagreement: the observed differences are compatible with the distinct operational definitions and methodological architectures of the ranking systems, but the rank-level analysis conducted here does not identify the indicator-level mechanisms responsible for those differences.

### 4.2 Institutional divergence as substantive information

The university-level trajectories provide further evidence that the ranking systems can portray the relative standing of the same institution differently. KSU and KFUPM represent the clearest contrasting cases. KSU was positioned substantially more strongly in ARWU than in QS or THE, reaching 90th globally in ARWU in 2024 while occupying 203rd place in QS and the 401–500 band in THE. KFUPM showed the reverse cross-ranking pattern in 2025, reaching 101st in QS and 176th in THE while remaining in the 701–800 band in ARWU.

These differences are sufficiently large to warrant substantive interpretation rather than being treated as minor positional variation. They demonstrate that the same institution can receive markedly different positional assessments across composite ranking systems. A plausible methodological interpretation is that differences in indicator selection, weighting, normalization, eligibility, and data treatment contribute to these divergent outputs. However, because the present study analyzes published ranks rather than indicator-level scores, it cannot determine which specific indicators or institutional characteristics caused the observed divergence. Cross-ranking disagreement should therefore be interpreted as evidence of system-dependent institutional positioning rather than as direct evidence of a distinctive underlying performance profile or an objectively uncertain “true rank.”

KAU provides an additional example. Its ARWU position moved from 101–150 in 2022 to 501–600 in 2025, while its QS position remained comparatively more stable. This difference supports earlier warnings against interpreting movements in a single ranking as a direct measure of underlying institutional improvement or decline. Alhuthali and Sayed (2022), in their examination of Saudi universities' ranking trajectories, similarly emphasized that ranking gains must be interpreted in relation to the indicators and institutional practices associated with the ranking system rather than assumed to represent broad-based institutional improvement.

The longitudinal interpretation also requires caution because ranking methodologies themselves change. THE introduced a major methodological revision in the 2024 edition, moving from 13 indicators organized around five areas to 18 indicators across five pillars. QS also introduced substantial methodological revisions for its 2024 edition. Consequently, part of the observed year-to-year movement may reflect changes in the measurement frameworks as well as changes in institutional performance or the composition and performance of the ranked population. This reinforces the need to distinguish changes in published rank from changes in underlying institutional quality.

Given the limited four-edition observation window and the absence of harmonized indicator-level data under both the pre-2024 and post-2024 methodologies, the independent effect of these methodological revisions cannot be formally isolated from concurrent changes in institutional performance and the ranked population. Accordingly, the 2022–2023 and 2024–2025 results are treated as descriptive temporal comparisons rather than as a methodologically homogeneous time series, and no causal interpretation is assigned to differences between the two periods.

### 4.3 . Rank aggregation produces consensus, but not a unique truth

The aggregation results provide a more nuanced picture than the cross-ranking comparisons alone. Borda, median-rank, and Copeland aggregation produced strongly correlated consensus solutions across all 4 years. This suggests that, within the year-specific common analytical sets, the broad relative ordering of the jointly ranked Saudi institutions was not highly dependent on the particular aggregation algorithm applied. Given the small number of institutions jointly represented across all three systems, however, this stability should be interpreted as a property of the observed common sets rather than generalized to Saudi universities as a whole.

This finding is important because rank aggregation is sometimes criticized on the grounds that it simply replaces one arbitrary league table with another. The present results indicate that, at least in this national case, a substantial component of the consensus ordering was robust across three genuinely different procedures. This is consistent with the broader literature showing that rank aggregation can provide a useful synthesis of heterogeneous league tables when methodological sensitivity is explicitly evaluated rather than ignored (Zhang et al., 2017; Salimova et al., 2021; Feng et al., 2023).

However, robustness at the overall level did not imply complete invariance. The most important exception occurred at the top of the 2025 consensus. Equal-weight Borda placed KSU first, whereas median-rank and Copeland placed KFUPM first. This difference is substantively meaningful because it directly reflects the conflict between ARWU and the other two systems. KFUPM led both QS and THE within the common set but performed much less strongly in ARWU, whereas KSU occupied the strongest ARWU position while ranking behind KFUPM in QS and THE.

This example demonstrates why an aggregated ranking should not be presented as a uniquely correct resolution of disagreement. The consensus outcome depends in part on what “consensus” is intended to mean. Borda rewards cumulative positional performance across all component rankings, median rank emphasizes the central position, and Copeland emphasizes pairwise majority superiority. When these methods agree, confidence in the structural stability of the combined ordering increases. When they disagree, that disagreement is itself evidence that the institutional comparison is sensitive to the normative logic of aggregation.

The decision not to treat mean rank as an independent alternative to Borda was also methodologically important. Under equal weights and a common complete set, the two are monotonic transformations and therefore do not provide genuinely independent evidence of robustness. Comparing structurally distinct methods provides a more meaningful test of aggregation sensitivity.

### 4.4 Cross-ranking profiles reveal what consensus conceals

The Pareto analysis provides perhaps the clearest demonstration of why aggregation alone is insufficient within the common analytical sets. The frontier expanded from a single institution in 2022 to three institutions—KAU, KSU, and KFUPM—in both 2024 and 2025. These institutions could not be unambiguously ordered across the three ranking systems because each exhibited a different cross-ranking profile. Importantly, this result applies only to the institutions jointly ranked by QS, THE, and ARWU in the relevant year and should not be interpreted as identifying the Pareto frontier of the Saudi higher education sector as a whole.

The 2025 profiles illustrate this particularly well. KFUPM occupied the strongest relative positions in QS and THE within the common analytical set, whereas KSU occupied the strongest relative position in ARWU. KAU showed a more balanced intermediate pattern across the three ranking outputs. None of these institutions could therefore be identified as consistently superior across all three systems, yet any scalar consensus ranking necessarily placed them in a linear order.

This result supports approaches that preserve multiple evaluation outputs rather than reducing them immediately to a single league-table position. Previous research has emphasized the value of performance profiles, partial-order approaches, and dashboard-based representations for reducing false precision in institutional evaluation (Matlis et al., 2024; Latifis et al., 2026). The present analysis applies a related logic to a policy-relevant national case by using Pareto dominance to preserve incomparability across three composite ranking outputs where a conventional consensus ranking would force a distinction. Importantly, the resulting cross-ranking profiles should not be interpreted as direct evidence of differences in underlying institutional dimensions such as teaching, research, or internationalization, because the analysis uses composite ranking outputs rather than indicator-level performance data.

The distinction between consensus aggregation and cross-ranking profile assessment is therefore important. Consensus addresses the practical question of how multiple ranking outputs may be summarized, whereas the Pareto analysis asks whether one institution occupies an equal or stronger relative position across all three ranking systems. These analyses provide complementary views of the observed ranking outputs, but neither establishes a uniquely correct ordering of underlying institutional quality.

From a policy perspective, Pareto non-dominance should therefore not be interpreted as evidence of higher institutional quality, overall excellence, or superior performance across the underlying missions of the university. It indicates only that, within the year-specific common analytical set, an institution is not simultaneously matched or exceeded by another institution across all three published ranking outputs. In the context of Vision 2030, the Pareto frontier can consequently serve as a diagnostic indicator of cross-ranking robustness: the presence of multiple non-dominated institutions signals that policy-relevant comparisons depend on which ranking system or aggregation rule is emphasized. Its value lies not in identifying a new “best” university or establishing an alternative policy target, but in making such system-dependent trade-offs explicit before ranking-based conclusions are used for institutional benchmarking or policy interpretation.

### 4.5 . Robustness to weighting and rank-band assumptions

The sensitivity analyses further qualify the interpretation of the consensus results. Under the three discrete asymmetric weighting scenarios, changes in institutional positions were generally small: all institutions remained within two positions of their equal-weight Borda baseline, and almost all remained within one position. This indicates that the broad consensus structure was relatively stable under moderate departures from equal weighting.

The continuous weight-space analysis, however, provides a more complete picture of this robustness by showing how the identity of the leading institution changes across the full range of admissible QS, THE, and ARWU weights. Leadership was highly stable in 2022, when KAU occupied the leading position throughout the interior of the weight space, and remained comparatively stable in 2023, when KAU led across approximately 88.9% of the admissible weight space. In 2024, leadership became more weight-dependent: KAU remained the leader across approximately 73.2% of the weight space, while KSU and KFUPM occupied smaller but substantive leadership regions. These results show that robustness cannot be assessed fully from a small number of pre-specified weighting scenarios alone.

The greatest sensitivity occurred in 2025. KSU led across approximately 53.3% of the admissible weight space and KFUPM across approximately 46.7%, with the transition between them determined by the relative emphasis assigned to ARWU vs. QS and THE. This near division of the weight space is substantively important because it shows that neither institution can be regarded as an overwhelmingly weight-robust consensus leader. Rather, KSU's leadership is supported by weighting combinations that give relatively greater influence to ARWU, whereas KFUPM becomes the leader as the combined influence of QS and THE increases. The result is consistent with the pronounced cross-ranking profile differences observed for the two institutions and demonstrates how aggregation can translate disagreement among ranking systems into sensitivity in the identity of the consensus leader.

These findings reinforce the distinction between robustness of the broader consensus structure and robustness of the leading position. Most institutional positions changed little under moderate reweighting, yet the identity of the leading university could remain sensitive across a substantial portion of the admissible weight space. This is consistent with Pinar et al. (2019), who demonstrated that university-ranking outcomes can be sensitive to weighting assumptions, and with Soh (2013), who cautioned against simplistic interpretations of nominal weights in composite rankings. In the present context, the continuous analysis makes this sensitivity explicit by identifying the regions of the weight space within which alternative institutional leaders emerge.

The deterministic rank-band sensitivity check produced no change in the equal-weight Borda ordering during 2022–2025 when the lower or upper endpoints of bounded intervals were substituted for the midpoint. This indicates that the observed consensus ordering was stable under the specific endpoint representations tested. However, this result should not be interpreted as evidence that uncertainty within published rank bands is inconsequential. Because endpoint values were converted into within-year ordinal positions before aggregation, relative ordering can remain unchanged even when the exact positions of institutions within published bands are unknown.

The contrast between the two sensitivity analyses should therefore be interpreted cautiously. Varying the relative influence assigned to QS, THE, and ARWU produced identifiable regions in which the consensus leader changed, whereas the tested lower-, midpoint-, and upper-endpoint representations did not alter the Borda ordering. The former demonstrates genuine sensitivity to the normative weighting of heterogeneous ranking outputs; the latter demonstrates only stability under the particular deterministic representations of bounded rank intervals examined here. A fuller treatment of within-band uncertainty would require interval-based or probabilistic aggregation methods that preserve the range of possible positions throughout the aggregation process.

### 4.6 Implications for vision 2030 and policy benchmarking

The policy-threshold analysis has direct implications for the use of global rankings under the Human Capability Development Program. The HCDP identifies the number of Saudi universities within the global top 100 and top 200 as policy indicators and explicitly references QS, THE, and Shanghai Ranking. The present results show that these nominally identical thresholds can yield materially different conclusions depending on the ranking system used.

The 2024 case is especially striking. ARWU placed KSU 90th globally, thereby identifying a Saudi institution within the top 100, while QS placed the same university just outside the top 200 and THE placed it in the 401–500 band. At the same time, QS identified KAU and KFUPM as top-200 institutions, while THE identified none. In 2025, QS identified three Saudi universities in the top 200, THE identified only KFUPM, and ARWU identified only KSU.

These differences do not imply that any provider reported its results incorrectly. Rather, they demonstrate policy-threshold uncertainty arising from ranking-system disagreement: the same nominal Top-100 or Top-200 criterion can produce different classifications of national achievement depending on the ranking system through which it is operationalized. The policy indicator is therefore instrument-dependent. A statement such as “Saudi Arabia has universities in the global Top 200” is incomplete unless the ranking system used to define that threshold is specified.

This finding has practical implications for policy monitoring. If national performance is evaluated through several ranking systems simultaneously, a transparent reporting framework should preserve system-specific results before presenting any combined interpretation. Using the most favorable ranking in a given year would risk selective reporting, whereas treating all systems as interchangeable would conceal substantial methodological differences. A more responsible approach would report the results of all three systems side by side, distinguish agreement from disagreement, and interpret progress over multiple years rather than through a single annual threshold observation.

At the institutional level, the framework can also function as a diagnostic monitoring tool. University leaders can first examine system-specific positions, then assess the extent to which those positions converge or diverge across rankings, and use the consensus and Pareto results to determine whether an institution's apparent standing is broadly robust or dependent on a particular ranking architecture. Substantial cross-system divergence can therefore serve as a diagnostic signal for further investigation of the underlying indicator-level performance rather than as evidence that one ranking necessarily provides the “correct” institutional position. In this sense, the framework helps identify when deeper institutional analysis is warranted, while stopping short of prescribing which individual indicators a university should optimize.

The 2024 KSU top-100 result also demonstrates why policy achievement and institutional trajectory should be distinguished. Crossing a threshold in one system is a legitimate observed result, but it does not necessarily imply that the institution has reached an equivalent position under other internationally recognized ranking frameworks. Rank-based policy indicators are therefore most informative when accompanied by explicit methodological context.

### 4.7 Implications for responsible research assessment

More broadly, the findings contribute to the debate over responsible university assessment. Gadd et al. (2021) argued that responsible use of rankings requires attention to transparency, methodological rigor, uncertainty, and the extent to which a ranking actually measures what stakeholders believe it measures. The present results provide empirical support for that argument.

Three forms of uncertainty emerged. The first was measurement-system uncertainty, reflected in disagreement among QS, THE, and ARWU. The second was aggregation uncertainty, reflected in limited but meaningful differences among Borda, median, and Copeland consensus rankings. The third was policy-classification uncertainty, reflected in different top-100 and top-200 conclusions across systems.

These forms of uncertainty should not be collapsed into one. A university may have a stable consensus rank despite highly divergent component profiles, and a stable relative position may still yield different policy classifications depending on the system. Responsible assessment therefore requires reporting both consensus and disagreement.

This point is particularly important because rankings can create an illusion of numerical precision. A rank such as 101, 176, or 200 appears exact, while a band such as 401–500 explicitly communicates uncertainty. Yet even exact ranks are conditional on the provider's indicator choices, data transformations, normalization procedures, and comparator population. The apparent precision of an ordinal position should therefore not be confused with measurement certainty.

### 4.8 Limitations and future research

Several limitations should be considered when interpreting the findings. First, the analysis covers only four annual editions, 2022–2025. This period was selected to maintain comparability across all three systems, but it limits inference about longer-term structural trends. Extending the analysis as future ARWU editions become available would allow stronger assessment of persistence and change.

Second, the analyses are constrained by the number of Saudi universities jointly represented across the ranking systems. The three-system common set used for consensus aggregation and Pareto assessment comprised only 4 institutions in 2022, 9 in 2023, 8 in 2024, and 9 in 2025. These complete-case analyses therefore characterize the subset of Saudi universities jointly ranked by QS, THE, and ARWU in each year and should not be generalized to the Saudi higher education sector as a whole. The same caution applies to pairwise positional correlations, particularly in years and ranking pairs with small numerical samples and tied rank bands. Accordingly, the reported Spearman and Kendall coefficients are interpreted as descriptive evidence of agreement within the observed common sets rather than as precise estimates of a broader population relationship. The complete-case restriction was retained because imputing ranks for institutions not published by a ranking provider would introduce artificial positional information.

Third, rank bands reduce positional precision, and the deterministic endpoint sensitivity analysis does not fully capture this uncertainty. Although lower-, midpoint-, and upper-bound representations produced the same Borda ordering in the analyses reported here, this stability should be interpreted cautiously because converting these representations into ordinal positions can preserve the relative ordering mechanically. The exact within-band positions of institutions remain unknown, and the present analysis does not evaluate all possible configurations within the published intervals. Future research could address this limitation through interval-based or probabilistic aggregation procedures that preserve positional uncertainty throughout the aggregation process rather than representing it through deterministic endpoint scenarios.

Fourth, methodological changes in QS and THE complicate longitudinal interpretation, particularly across the 2023–2024 boundary. With only four annual editions, the present study cannot reliably disentangle changes associated with ranking methodology from changes in institutional performance, the composition of the ranked population, or other year-specific factors. The descriptive distinction between 2022 and 2023 and 2024–2025 should therefore not be interpreted as a formal pre/post evaluation of the methodological revisions. Future research using longer observation windows could examine periods before and after major methodological reforms more systematically and assess whether observed positional shifts persist beyond the transition editions.

Fifth, this study analyzes published ranks rather than underlying indicator-level scores. This was intentional because the purpose was to develop a framework for monitoring and interpreting policy-relevant ranking outputs rather than an optimization model for prescribing institutional improvement strategies. Consequently, the framework can identify where institutional conclusions are robust or system-dependent and where deeper diagnostic analysis may be warranted, but it cannot determine which specific indicators an institution should prioritize to improve its position. Such decisions would require a different analytical design based on indicator-level data, institutional priorities, and the specific measurement architecture of each ranking system. Future research could therefore connect the present rank-level framework with indicator-level multidimensional models and improvement scenarios where sufficiently comparable provider data are available.

Future research could also extend the framework beyond Saudi Arabia to other countries whose national higher education strategies use global ranking targets. Comparative analyses across Gulf Cooperation Council states or other emerging higher education systems would help determine whether the patterns observed here are specific to Saudi institutional structures or represent a broader consequence of using heterogeneous global rankings for national policy evaluation. Incorporating mission-sensitive indicators of teaching, societal impact, innovation, sustainability, and regional contribution could further test whether international ranking performance aligns with broader conceptions of university quality and public value.

## 5 Conclusions

This study examined whether the international standing of Saudi universities can be interpreted more robustly by combining evidence from QS, THE, and ARWU rather than relying on a single global league table. Using year-aligned data for 2022–2025, the analysis integrated cross-ranking agreement, alternative rank-aggregation procedures, cross-ranking profile assessment, and sensitivity testing within the policy context of Saudi Vision 2030.

The findings show that the three ranking systems provide related but non-equivalent representations of institutional standing. QS and THE generally showed stronger positional agreement, whereas ARWU frequently produced substantially different institutional orderings. These differences were particularly evident for leading Saudi universities such as KSU and KFUPM and were sufficiently large to affect both institutional comparisons and policy-relevant Top-100 and Top-200 classifications. Accordingly, the three systems should not be treated as interchangeable measures of international university standing.

At the same time, Borda, median-rank, and Copeland aggregation produced broadly consistent consensus structures. The consensus ordering was stable under the deterministic rank-band endpoint specifications tested and showed limited positional sensitivity under the alternative system-weight scenarios examined. These results suggest that rank aggregation can provide a useful summary of multiple ranking outputs within the observed common sets, while also demonstrating that a single consensus ordering does not preserve all information contained in cross-ranking disagreement. The Pareto analysis illustrates this distinction: KAU, KSU, and KFUPM were non-dominated within the common analytical sets in both 2024 and 2025 because none occupied an equal or stronger relative position than the others across all three ranking systems. This finding reflects differences in their cross-ranking positional profiles and should not be interpreted as direct evidence of distinct underlying dimensions of institutional performance.

The policy implications are particularly relevant because the Human Capability Development Program references QS, THE, and Shanghai Ranking in assessing Saudi universities' international standing. The same Top-100 or Top-200 threshold can produce different counts and identify different universities depending on the ranking system applied. Policy monitoring should therefore report system-specific outcomes transparently and interpret threshold achievement in conjunction with cross-ranking consistency rather than reducing national performance to an unspecified single league-table result.

Overall, the study supports a shift from ranking-centered evaluation to evidence-centered interpretation of rankings. Moving beyond league tables does not require abandoning global university rankings; rather, it requires recognizing their methodological plurality, testing the sensitivity of conclusions across systems and aggregation assumptions, and preserving cross-system differences where a single consensus rank would create false precision. The practical value of the proposed framework lies primarily in supporting transparent monitoring, diagnostic benchmarking, and interpretation of ranking-based policy outcomes, rather than in prescribing indicator-level strategies for improving institutional positions. For Saudi higher education—and potentially for other national systems that incorporate global rankings into strategic targets—the combined use of cross-ranking comparison, rank aggregation, and cross-ranking profile assessment can help distinguish relatively stable conclusions from system-dependent ones and identify when more detailed indicator-level investigation is warranted.

## Data Availability

The data analyzed in this study were obtained from publicly available QS World University Rankings, Times Higher Education World University Rankings, and Academic Ranking of World Universities (ShanghaiRanking) for the 2022–2025 editions. The relevant findings and supporting information are included in the article. Further inquiries regarding the data and analytical procedures may be directed to the corresponding author.
